# A novel MLO protein CsMLO4 plays an essential role in cucumber resistance to target leaf spot

**DOI:** 10.1093/hr/uhaf225

**Published:** 2025-09-10

**Authors:** Yongbo Yu, Xiangnan Meng, Yang Yu, Jiajing He, Yuying Jiang, Jinghang Hong, Na Cui, Run Cai, Jan Pan, Junsong Pan, Haiyan Fan

**Affiliations:** College of Bioscience and Biotechnology, Shenyang Agricultural University, Shenyang 110866, China; College of Bioscience and Biotechnology, Shenyang Agricultural University, Shenyang 110866, China; Key Laboratory of Protected Horticulture of Ministry of Education, Shenyang Agricultural University, Shenyang 110866, China; College of Bioscience and Biotechnology, Shenyang Agricultural University, Shenyang 110866, China; Key Laboratory of Protected Horticulture of Ministry of Education, Shenyang Agricultural University, Shenyang 110866, China; College of Bioscience and Biotechnology, Shenyang Agricultural University, Shenyang 110866, China; College of Bioscience and Biotechnology, Shenyang Agricultural University, Shenyang 110866, China; College of Bioscience and Biotechnology, Shenyang Agricultural University, Shenyang 110866, China; College of Bioscience and Biotechnology, Shenyang Agricultural University, Shenyang 110866, China; Key Laboratory of Protected Horticulture of Ministry of Education, Shenyang Agricultural University, Shenyang 110866, China; College of Agriculture and Biology, Shanghai Jiao Tong University, Shanghai 200240, China; College of Horticulture, Shenyang Agricultural University, Shenyang 110866, China; College of Agriculture and Biology, Shanghai Jiao Tong University, Shanghai 200240, China; College of Bioscience and Biotechnology, Shenyang Agricultural University, Shenyang 110866, China; Key Laboratory of Protected Horticulture of Ministry of Education, Shenyang Agricultural University, Shenyang 110866, China

## Abstract

Target leaf spot (TLS), caused by *Corynespora cassiicola*, is a prevalent leaf disease that significantly impacts cucumber yield and quality. Breeding disease-resistant cucumber varieties is a key strategy for managing this disease, and identifying critical resistance genes is essential for genetic improvement. In this study, we identified a highly susceptible mutant to TLS in the *Tnt1* retrotransposon mutant library. Bulked segregation analysis sequencing (BSA-seq) further pinpointed a candidate gene for TLS resistance, encoding the Mildew Resistance Locus O (MLO) protein, *CsMLO4*. Expression analysis revealed that *CsMLO4* is strongly induced by *C. cassiicola* infection. Functional analyses revealed that loss of function and silencing of *CsMLO4* attenuated resistance to TLS and exhibited reduced reactive oxygen species (ROS) accumulation, while transient overexpression of *CsMLO4* enhanced both disease resistance and ROS levels. These findings suggest that *CsMLO4* mediates cucumber defense against *C. cassiicola* by modulating ROS levels. Additionally, transcriptome analysis identified multiple disease-resistance-related pathways affected by the loss of function of *CsMLO4*. Overexpression of *CsMYB*, a potential candidate gene regulated by *CsMLO4*, showed enhanced resistance to *C. cassiicola*. This study expands insights into the functional role of *MLO* family beyond their association with powdery mildew resistance and offers new perspectives on the mechanisms underlying TLS resistance in cucumber.

## Introduction

Cucumber (*Cucumis sativus* L.) is an economic vegetable crop that is widely cultivated worldwide [1, 2]. Target leaf spot (TLS), caused by the fungal pathogen *Corynespora cassiicola*, is a destructive leaf disease in cucumber cultivation, severely limiting the sustainable production of cucumbers [3]. At present, the application of fungicides is considered the main strategy for controlling TLS. However, continuous high-dose and high-frequency use of fungicides has resulted in resistance to *C. cassiicola*, and the application of these chemical fungicides also poses a serious threat to human health and the environment [4, 5]. Therefore, it is necessary and urgent to cultivate TLS-resistant cucumber varieties by mining new resistance genes and exploring their genetic and molecular mechanisms of defense.

Mildew Resistance Locus O (MLO) is a plant-specific protein containing seven conserved transmembrane domains and one calmodulin-binding domain located at the C-terminus [6]. The MLO gene family has been a focus of extensive research because of the broad-spectrum resistance to powdery mildew produced by gene mutations. The susceptibility of MLO to powdery mildew was first identified in barley, where the recessive loss-of-function allele, *Hvmlo*, imparts broad-spectrum and durable resistance to powdery mildew [7, 8]. In recent years, with the availability of more and more plant reference genomes, the function of barley *HvMLO* homologous genes as susceptible members for powdery mildew has been successively demonstrated in other species, such as *Arabidopsis thaliana* (*AtMLO2*, *AtMLO6*, and *AtMLO12*) [9], tomato (*SlMLO1*) [10], wheat (*TaMLO-A1*, *TaMLO-B1*, and *TaMLO-D1*) [11, 12], and cucumber (*CsMLO1*, *CsMLO8*, and *CsMLO11*) [13]. In addition, other disease resistance pathways, apart from powdery mildew, that MLO regulates have also garnered increasing attention. Heterologous expression of the oilseed rape *BnMLO2_2* gene in *A. thaliana* enhanced the resistance of transgenic plants to *Sclerotinia* stem rot [14]. In chili pepper, the immune regulation of CaMLO1 against *Ralstonia solanacearum* (bacterial wilt) is affected by temperature; specifically, it is positive against *R. solanacearum* at 37°C, but negative at 28°C [15]. Wheat *Tamlo* mutant increases susceptibility to *Magnaporthe oryzae* pv. *Triticum* (blast disease) [16]. Thus, MLO is involved in the defense regulation of a range of different pathogens and is an important member of plant immunity; however, the biological functions and genetic and molecular mechanisms conferred by most MLO remain largely unknown.

Several studies have been conducted to explore the possibility of *MLO* genes regulating disease resistance in plants. In barley and *A. thaliana*, *mlo*-mediated immunity is closely associated with enhanced callose deposition in the cell wall [17–19]. Recently, MLO proteins have been revealed to function as Ca^2+^-permeable channels [20]. In rice, MLO acts as a novel calmodulin-binding protein that can be considered a player in plant defense and cell death [21]. Additionally, *mlo* mutants are accompanied by spontaneous accumulation of reactive oxygen species (ROS) and salicylic acid (SA) [9, 22]. ROS is a regulatory factor and signal transduction molecule involved in multiple cellular pathways and plays a crucial role in plant immune response to pathogen attack [23–25]. Rapid accumulation of ROS at the site of pathogen infestation can limit pathogen growth, which in turn prompts a hypersensitive response (HR) in plants [26, 27]. HR can activate signaling pathways such as SA and defense genes such as pathogenesis-related (PR) genes, enabling plants to develop systemic acquired resistance (SAR) [28–31]. Notably, excessive accumulation of ROS can cause oxidative damage and toxicity to plant cells [32]. Therefore, maintaining the dynamic balance of ROS in plants is crucial.

**Figure 1:**
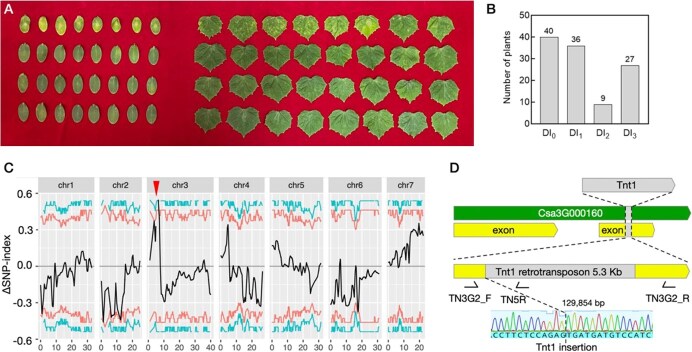
Identification of target leaf spot (TLS) resistance gene in cucumber. (A) Identification of TLS resistance of the M_2_ population. (B) Frequency distribution of disease index (DI) scores in population. DI_0_, 0 < DI ≤ 25; DI_1_, 25 < DI ≤ 50; DI_2_, 50 < DI ≤ 75; DI_3_, DI > 75. (C) BSA of TLS susceptibility locus based on SNP-index algorithm. The Manhattan plot of ∆ (SNP-index). The red line is the fitted ∆ (SNP-index). The green and orange lines indicate the thresholds of the top 5% and 1%, respectively. The confidence interval of TLS susceptibility locus limited with red dotted lines in 2.8 Mb at chromosome 3. (D) Structure of the candidate gene *Csa3G000160* (*CsMLO4*). A 5.3-kb *Tnt1* retrotransposon was inserted into the second exon of *Csa3G000160*. To identify the insert effect, primers, namely TN3G2_F and TN3G2_R, were designed based on the flanking sequence, along with primer TN5R within the *Tnt1* insertion.

Much progress has been made in resolving the function of MLO and ROS in plant immunity. However, it is still largely unknown whether MLO is involved in regulating resistance to *C. cassiicola* and whether it mediates immunity through the ROS pathway in cucumber. Here, we identified a mutant that is highly susceptible to TLS by inoculating *C. cassiicola* with a *Tnt1* retrotransposon insertion mutant collection. Further bulked segregation analysis sequencing (BSA-seq) revealed the presence of only a unique *Tnt1* insertion event within the mapping interval, anchored to the *Csa3G000160* gene. *Csa3G000160* encodes an MLO protein, named *CsMLO4*. Analysis of mutant, transient silencing, and overexpression plants showed that *CsMLO4* positively regulated the resistance to *C. cassiicola* and ROS accumulation. Furthermore, multiple genes and multiple biological processes in *C. cassiicola* defense mediated by *CsMLO4* were identified by RNA sequencing (RNA-seq). *CsMYB*, a potential candidate regulated by *CsMLO4*, was shown to be a positive regulator of *C. cassiicola* resistance. These results provide a new perspective for studying the molecular mechanism of cucumber defense response to TLS, as well as a new genetic resource for cultivating TLS-resistant cucumber varieties.

## Results

### *CsMLO4* governs the resistance to cucumber TLS

To uncover key regulators that control resistance to TLS in cucumber, we screened the *Tnt1* retrotransposon insertion mutant collection of cucumber through inoculation with the *C. cassiicola* pathogen. We found a mutant in the M_2_ (24TN902 line) segregating population that exhibits high susceptibility to TLS (Fig. 1A). We categorized the disease severity into four disease indexes (DI_0_ to DI_3_) in the M_2_ segregating population. By investigating the resistance in the segregating population, we observed a trough at DI_2_, with a bimodal distribution of resistance levels (Fig. 1B). Although resistance to *C. cassiicola* in natural cucumber populations is typically a quantitatively inherited trait involving multiple genes, the mutant in this study was identified from a *Tnt1* insertion mutant library, in which phenotypic variation is more commonly attributed to loss-of-function mutations in single genes. In line with this, the observed distribution of DI was skewed toward the resistant type, suggesting that the loss of TLS resistance was likely due to the disruption of a single gene by *Tnt1* insertion. To further confirm the loss of TLS resistance in the mutant, we crossed the susceptible mutant 24TN902 with the resistant inbred line S06. The segregation ratio of resistant to susceptible plants in the progeny was approximately 3:1 (Table S1), indicating that the hypersusceptible phenotype of the mutant was caused by a single recessive gene mutation.

**Figure 2:**
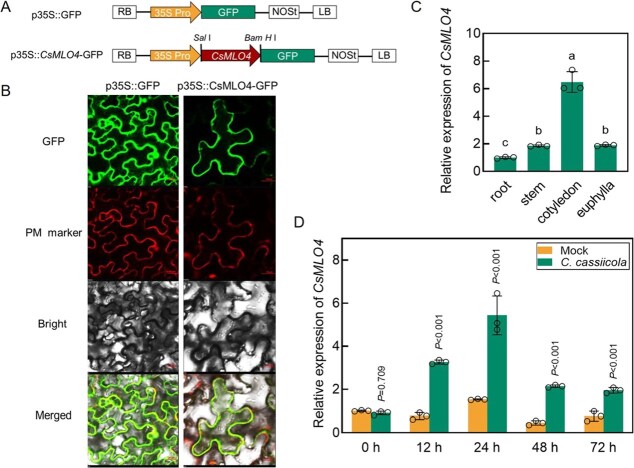
Expression analysis of *CsMLO4*. (A) Schematic of p35S::GFP and p35S::*CsMLO4*-GFP vectors. (B) Subcellular localization of CsMLO4 in *N. benthamiana* cells. The fusion protein p35S::CsMLO4-GFP was co-expressed with a plasma membrane (PM) marker in *N. benthamiana* and visualized using a laser confocal microscope. The p35S::GFP with PM marker co-permeabilizer was employed as a control. Bars, 20 μm. (C) Expression analysis of *CsMLO4* in different tissues of cucumber. (D) Expression analysis of *CsMLO4* at different time points following inoculation of cucumbers with *C. cassiicola*. Mock, untreated control. (C, D) Data are means ± SD of three biological replicates per variety. Significance was assessed by the Duncan (*P* ≤ 0.05) and LSD multiple comparison test.

Subsequently, we employed BSA-seq to locate the candidate gene that was responsible for the loss of TLS resistance. After calculating statistical confidence intervals of *P* < 0.01 between the two extreme phenotypic bulks, the SNP-index calculation identified a significant signal at one end of chromosome 3 (Fig. 1C), where only one *Tnt1* insertion event occurred near the locus within the mapping interval. The *Tnt1* retrotransposon was inserted into the second exon of the *Csa3G000160* gene, leading to loss of function of this gene (Fig. 1D). *Csa3G000160* encodes an MLO protein. Additional allelic mutants TN183-2 exhibited identical phenotypes (Fig. S1), and allelism tests confirmed that *Csa3G000160* underlies the resistance to *C. cassiicola*. Based on previous studies on *MLO* genes in cucumber [13], we named this candidate gene *CsMLO4*.

### Expression analysis of *CsMLO4*

Sequence analysis of *CsMLO4* revealed that the gene contains a 1710-bp coding sequence (CDS) encoding 569 amino acids, seven transmembrane domains, and an MLO domain (Fig. S2). Phylogenetic analysis showed that within the Cucurbitaceae family, CsMLO4 shows a close evolutionary relationship with homologs from *Cucumis melo*, *Citrullus colocynthis*, and *Benincasa hispida*, highlighting strong conservation of CsMLO4. Similarly, homologs in the Solanaceae family, such as those in *Solanum stenotomum*, *Nicotiana sylvestris*, and *Capsicum annuum*, form their own distinct cluster, reflecting a degree of divergence from Cucurbitaceae. In contrast, homologs from the Rutaceae family, such as those in *Citrus sinensis* and *Citrus unshiu*, and cereal crops, including *Zea mays* and *Oryza sativa*, exhibit greater genetic distances (Fig. S3).

Further, subcellular localization showed that CsMLO4, when fused with green fluorescent protein (GFP), localized to the plasma membrane (PM) (Fig. 2A and B), which matched our prediction and indicated that CsMLO4 was a typical membrane protein. To investigate the spatial expression pattern of *CsMLO4*, we measured its relative expression in different tissues of cucumber and showed that *CsMLO4* was expressed predominantly in cotyledons, followed by stems and euphylla, and the lowest in the roots (Fig. 2C). Furthermore, we analyzed the expression levels of *CsMLO4* in cucumbers infected with *C. cassiicola* at different times to explore the temporal expression pattern of *CsMLO4*. The results showed that *CsMLO4* was significantly induced by *C. cassiicola*, indicating that *CsMLO4* is a participant in *C. cassiicola* resistance (Fig. 2D).

### Loss of function of *CsMLO4* inhibits resistance to *C. cassiicola* and ROS accumulation

**Figure 3:**
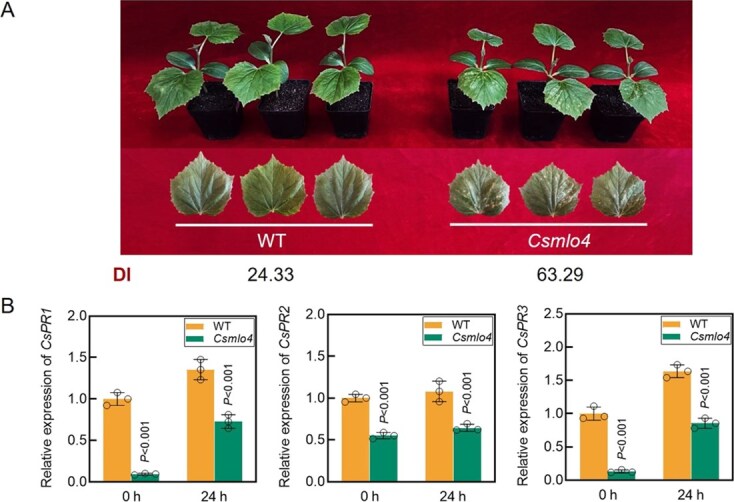
Loss of function of *CsMLO4* enhances the susceptibility of cucumber to *C. cassiicola*. (A) Disease resistance of WT and loss of function of *CsMLO4* mutant line 24TN902 (*Csmlo4*) leaves were assessed using phenotypic symptoms and DI statistics at 7 days postinoculation (dpi) with *C. cassiicola*. (B) Expression levels of pathogenesis-related genes *CsPR1*, *CsPR2*, and *CsPR3* in WT and *Csmlo4* plants before (0 hours) and after (24 hours) infection with *C. cassiicola.* Data are means ± SD of three biological replicates per variety. Significance was assessed by the LSD multiple comparison test.

Upon *C. cassiicola* inoculation, loss of function of *CsMLO4* mutant line (*Csmlo4*) plants displayed more severe TLS lesions and higher DI than the wild type (WT) (Fig. 3A), suggesting that loss of function of *CsMLO4* compromises resistance. Additionally, the expression of three defense-related genes *CsPR1*, *CsPR2*, and *CsPR3* was markedly reduced in *Csmlo4* plants (Fig. 3B), indicating that *CsMLO4* positively regulates their expression during defense. Therefore, we speculate that *CsMLO4* positively regulates defense against *C. cassiicola* through the promotion of *CsPR1*, *CsPR2*, and *CsPR3* expression.

**Figure 4:**
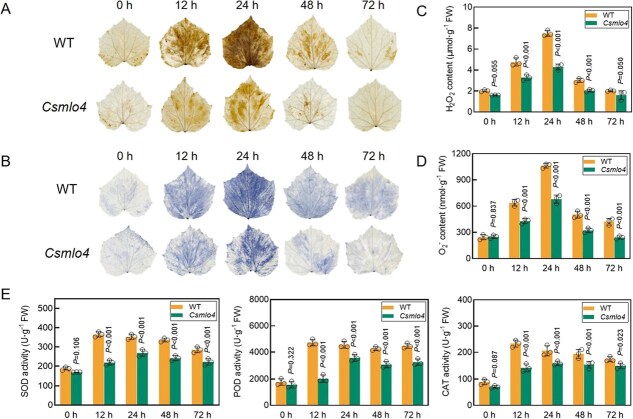
Loss of function of *CsMLO4* inhibits ROS accumulation. (A, B) DAB (A) and NBT (B) staining of WT and *Csmlo4* plants inoculated with *C. cassiicola*. (C, D) Determination of H_2_O_2_ (C) and O_2_^−^ (D) contents in WT and *Csmlo4* plants inoculated with *C. cassiicola*. (E) Determination of superoxide dismutase (SOD), peroxidase (POD), and catalase (CAT) activities in WT and *Csmlo4* plants inoculated with *C. cassiicola*. (C–E) FW stands for fresh weight. Data are means ± SD of three biological replicates per variety. Significance was assessed by the LSD multiple comparison test.

ROS accumulation is one of the rapidly formed defense responses of plants to pathogen stress *in vivo*. To explore the role of *CsMLO4* in ROS-mediated defenses, H_2_O_2_ and O_2_^−^ were assessed by 3,3′-diaminobenzidine (DAB) and p-nitroblue tetrazolium chloride (NBT) staining. Staining intensity peaked at 24 hours postinoculation (hpi) in both genotypes but was weaker in *Csmlo4* (Fig. 4A and B). Consistently, H_2_O_2_ and O_2_^−^ contents in *Csmlo4* were significantly lower than in WT during early infection stages (Fig. 4C and D). The activities of superoxide dismutase (SOD), peroxidase (POD), and catalase (CAT) peaked at 12 hpi in WT but were delayed to 24 hpi in *Csmlo4*, with significantly reduced overall activity levels (Fig. 4E). These results demonstrate that *CsMLO4* enhances cucumber resistance to *C. cassiicola* by promoting ROS accumulation, timely antioxidant enzyme activation, and defense gene expression.

### Transient overexpression of *CsMLO4* promotes defense against *C. cassiicola* and ROS accumulation

**Figure 5:**
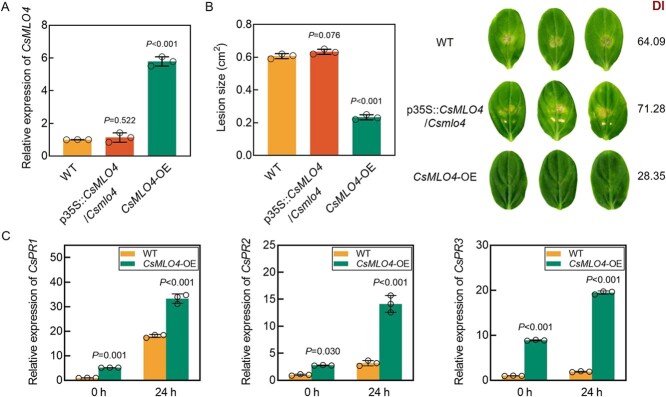
Overexpression of *CsMLO4* increases the resistance of cucumber to *C. cassiicola*. (A) Expression levels of *CsMLO4* in WT, p35S::*CsMLO4*/*Csmlo4*, and overexpression (*CsMLO4*-OE) cucumbers*.* (B) Disease resistance of WT, p35S::*CsMLO4*/*Csmlo4*, and *CsMLO4*-OE cucumber cotyledons was assessed using phenotypic symptoms, DI statistics, and lesion size at 5 dpi with *C. cassiicola*. (C) Expression levels of *CsPR1*, *CsPR2*, and *CsPR3* in WT and *CsMLO4*-OE cucumbers before (0 hours) and after (24 hours) infection with *C. cassiicola.* (A–C) Data are means ± SD of three biological replicates per variety. Significance was assessed by the LSD multiple comparison test.

To confirm the role of *CsMLO4* in *C. cassiicola* resistance, we introduced the overexpression vector p35S::*CsMLO4*-GFP into the cotyledons of *Csmlo4* and WT cucumber seedlings, respectively, and constructed p35S::*CsMLO4*/*Csmlo4* and overexpression (*CsMLO4*-OE) plants. The expression of *CsMLO4* returned to the WT level in p35S::*CsMLO4*/*Csmlo4* plants and significantly increased in *CsMLO4*-OE plants (Fig. 5A). The symptoms, DI, and lesion size of p35S::*CsMLO4*/*Csmlo4* plants were similar to WT. In contrast, *CsMLO4*-OE plants exhibited higher resistance, i.e. milder symptoms, lower DI, and smaller lesion size than WT plants at 5 days postinoculation (dpi) with *C. cassiicola* (Fig. 5B). These data provide further evidence that *CsMLO4* is a positive responder of the cucumber defense against *C. cassiicola*. Next, we analyzed *CsPR1*, *CsPR2*, and *CsPR3* expression levels in WT and *CsMLO4*-OE plants before and after infection with *C. cassiicola*. The results indicated that overexpression of *CsMLO4* remarkably promoted the expression of *CsPR1*, *CsPR2*, and *CsPR3* (Fig. 5C), verifying the positive regulatory effect of *CsMLO4* on *CsPR1*, *CsPR2*, and *CsPR3*.

**Figure 6:**
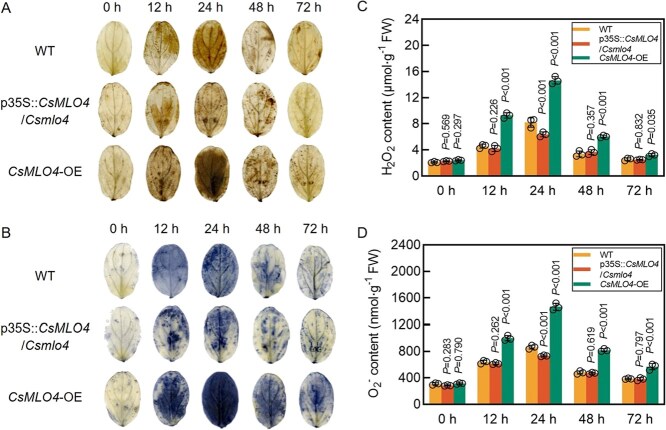
Overexpression of *CsMLO4* promotes ROS accumulation. (A, B) DAB (A) and NBT (B) staining of WT, p35S::*CsMLO4*/*Csmlo4*, and *CsMLO4*-OE cucumber cotyledons inoculated with *C. cassiicola*. (C, D) Determination of H_2_O_2_ (C) and O_2_^−^ (D) contents in WT, p35S::*CsMLO4*/*Csmlo4*, and *CsMLO4*-OE cucumber cotyledons inoculated with *C. cassiicola*. FW stands for fresh weight. Data are means ± SD of three biological replicates per variety. Significance was assessed by the LSD multiple comparison test.

DAB and NBT staining of WT, p35S::*CsMLO4*/*Csmlo4*, and *CsMLO4*-OE plant cotyledons infected with *C. cassiicola* demonstrated that the areas of brown and blue spots on leaves exhibited a tendency to increase and then decrease as the duration of inoculation with *C. cassiicola* was extended, peaking at 24 hpi. Compared with WT, p35S::*CsMLO4*/*Csmlo4* plants presented similar phenotypes, while brown and blue complexes appeared earlier and in larger areas in *CsMLO4*-OE cucumber cotyledons, suggesting faster and more accumulation of H_2_O_2_ and O_2_^−^ (Fig. 6A and B). In addition, detection of H_2_O_2_ and O_2_^−^ contents in WT, p35S::*CsMLO4*/*Csmlo4*, and *CsMLO4*-OE cucumber cotyledons infected with *C. cassiicola* yielded results consistent with histochemical staining; i.e. overexpression of *CsMLO4* increased H_2_O_2_ and O_2_^−^ contents during the early defense response of cucumber to *C. cassiicola* (Fig. 6C and D). The results suggest that *CsMLO4* may enhance defense against *C. cassiicola* by promoting ROS accumulation. Furthermore, the expression levels of *CsMLO4* in WT, p35S::*CsMLO4*/*Csmlo4*, and *CsMLO4*-OE plants at 0–72 hpi also indicated that the changes in ROS levels during *C. cassiicola* infection were closely associated with the changes in *CsMLO4* expression (Fig. S4).

### Transient silencing of *CsMLO4* attenuates defense against *C. cassiicola* and ROS accumulation

**Figure 7:**
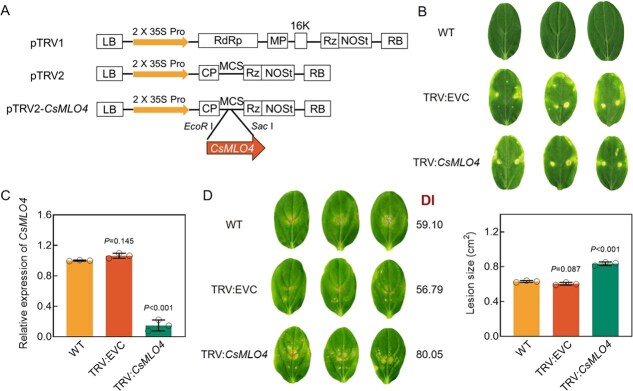
Silencing of *CsMLO4* attenuates the resistance of cucumber to *C. cassiicola*. (A) Schematic diagram of the silent expression constructs. (B) Phenotype of symptoms of TRV invasion in cucumber. (C) Expression levels of *CsMLO4* in WT, TRV:EVC, and TRV:*CsMLO4* cucumbers*.* (D) Disease resistance of WT, TRV:EVC, and TRV:*CsMLO4* cucumber cotyledons was assessed using phenotypic symptoms, DI statistics, and lesion size at 5 dpi with *C. cassiicola*. (C, D) Data are means ± SD of three biological replicates per variety. Significance was assessed by the LSD multiple comparison test. (B–D) EVC stands for empty vector control.

To further clarify the function of *CsMLO4* in cucumber defense against *C. cassiicola*, we silenced *CsMLO4* in cucumber using tobacco rattle virus (TRV)-mediated virus-induced gene silencing (VIGS). A specific fragment of *CsMLO4* CDS was inserted into the pTRV2 vector to create pTRV2-*CsMLO4* construct (Fig. 7A). The chlorosis mosaic symptoms on the cotyledons of TRV:EVC and TRV:*CsMLO4* cucumbers indicated that TRV had successfully invaded the plants, which was not observed in WT plants (Fig. 7B). The expression level of *CsMLO4* was significantly reduced in silenced plants (Fig. 7C). Five days postinfection with *C. cassiicola*, *CsMLO4*-silenced plants showed more severe symptoms, larger lesion size, and higher DI compared to WT and TRV:EVC plants (Fig. 7D). In addition, similar results were obtained by silencing *CsMLO4* based on cucumber green mottle mosaic virus (CGMMV)-mediated VIGS, i.e. *CsMLO4*-silenced plants showed enhanced susceptibility compared to pV190:EVC at 5 dpi (Fig. S5).

**Figure 8:**
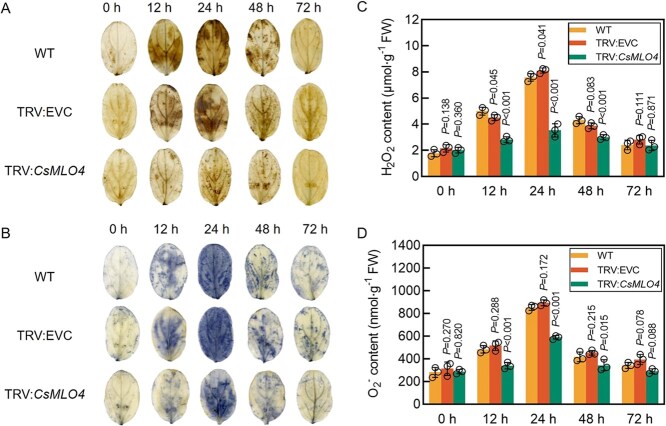
Silencing of *CsMLO4* inhibits ROS accumulation. (A, B) DAB (A) and NBT (B) staining of WT, TRV:EVC, and TRV:*CsMLO4* cucumber cotyledons inoculated with *C. cassiicola*. (C, D) Determination of H_2_O_2_ (C) and O_2_^−^ (D) contents in WT, TRV:EVC, and TRV:*CsMLO4* cucumber cotyledons inoculated with *C. cassiicola*. FW stands for fresh weight. Data are means ± SD of three biological replicates per variety. Significance was assessed by the LSD multiple comparison test. EVC stands for empty vector control.

Histochemical staining and content determination of H_2_O_2_ and O_2_^−^ were performed on the cotyledons of WT, TRV:EVC, and TRV:*CsMLO4* cucumbers inoculated with *C. cassiicola*. As depicted in Fig. 8, with the progression of *C. cassiicola* infection over time, the accumulation of H_2_O_2_ and O_2_^−^ on cucumber leaves exhibited a trend of initially increasing and subsequently decreasing, peaking at 24 hpi. Compared with WT and TRV:EVC plants, the levels of H_2_O_2_ and O_2_^−^ in the cotyledons of *CsMLO4*-silenced cucumbers were significantly reduced. These findings suggest that the silencing of *CsMLO4* suppresses the accumulation of ROS, consequently diminishing the resistance of cucumbers to *C. cassiicola*.

### Multiple genes and multiple biological processes function in *CsMLO4*-mediated defense against *C. cassiicola*

**Figure 9:**
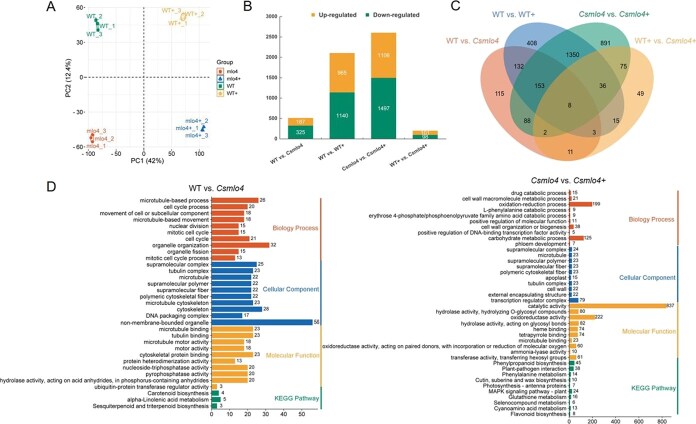
RNA sequencing (RNA-seq) analysis of WT and *Csmlo4* plants before and after inoculation with *C. cassiicola*. (A) PCA of samples. 1, 2, and 3 represent three biological replicates of the sample. (B) The number of DEGs obtained in each comparison group. (C) Venn diagrams of DEGs in each comparison group. (D) Gene Ontology (GO) and KEGG analyses of DEGs in the WT vs *Csmlo4* and *Csmlo4* vs *Csmlo4*+ comparison groups. (A–D) ‘+’ means that the sample was infected with *C. cassiicola* for 24 hours.

**Figure 10:**
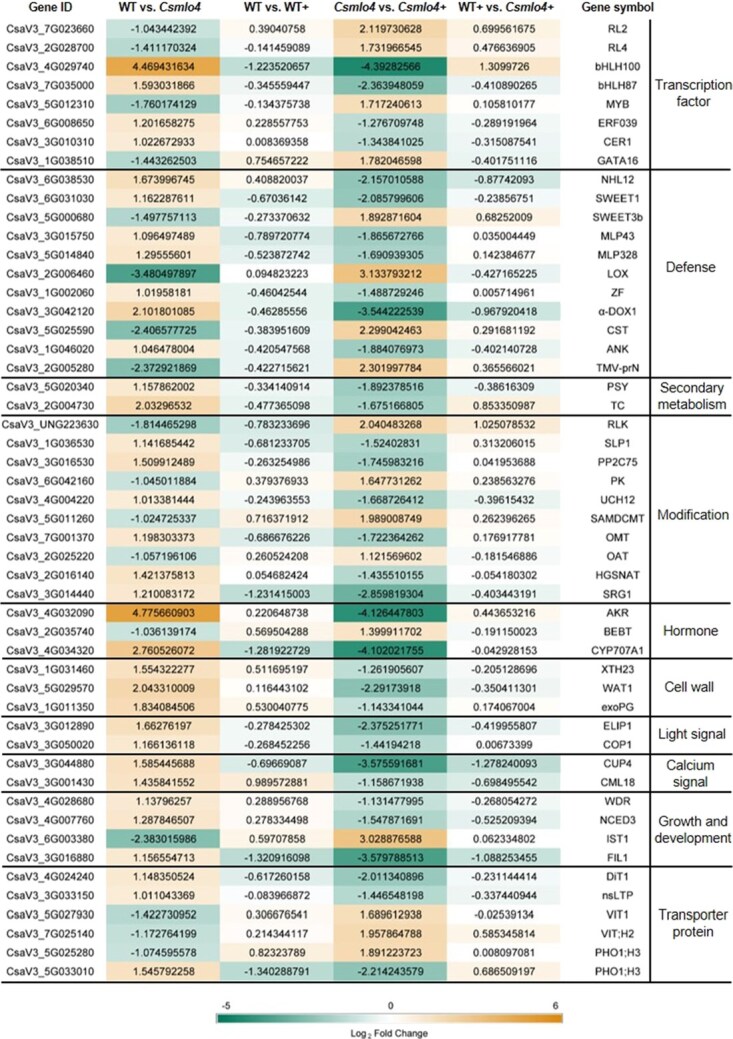
Heatmap analysis of candidate genes regulated by *CsMLO4* during *C. cassiicola* defense. The color scale from green (low) to orange (high) indicates the log_2_FoldChange of the transcripts. ‘+’ means that the sample was infected with *C. cassiicola* for 24 hours.

To mine key regulatory genes of *CsMLO4*-mediated cucumber resistance to *C. cassiicola*, we performed RNA-seq analyses on WT and *Csmlo4* plants inoculated or uninoculated with *C. cassiicola*. Principal component analysis (PCA) confirmed the high quality and consistency of the RNA-seq data (Fig. 9A). A total of 3336 differentially expressed genes (DEGs) were found between the WT and *Csmlo4* (WT vs *Csmlo4*) group, between the WT and WT inoculated with *C. cassiicola* (WT vs WT+) group, between the *Csmlo4* and *Csmlo4* inoculated with *C. cassiicola* (*Csmlo4* vs *Csmlo4*+) group, and between the WT inoculated with *C. cassiicola* and *Csmlo4* inoculated with *C. cassiicola* (WT+ vs *Csmlo4*+) group. For the WT vs *Csmlo4* group, a total of 512 DEGs were found, of which 187 were up-regulated and 325 were down-regulated. After *C. cassiicola* infection, 2105 DEGs (965 up-regulated and 1140 down-regulated) were found in the WT group, and 2603 DEGs (1106 up-regulated and 1497 down-regulated) were found in the *Csmlo4* group (Fig. 9B and C). One thousand five hundred forty-seven DEGs in both groups were co-regulated under *C. cassiicola* infection, whereas 558 DEGs were exclusive to the WT group and 1056 DEGs were exclusive to the *Csmlo4* group, implying that loss of function of *CsMLO4* mobilized additional molecular players to mediate the immune response to *C. cassiicola*. Notably, 90 of these DEGs present in the *Csmlo4* group but not in the WT group were shared with the WT vs *Csmlo4* group, which may be key regulatory genes in the *CsMLO4*-mediated *C. cassiicola* resistance pathway, and are mainly associated with cell wall, secondary metabolism, light signals, calcium signals, hormones, growth and development, DNA/protein modifications, transporter proteins, transcription factors, and defense responses (Figs 9C and 10; Table S2). We randomly selected 16 of these 90 DEGs for expression analysis, and the high consistency between quantitative reverse transcription polymerase chain reaction (qRT-PCR) and RNA-seq results validated the reliability of the RNA-seq (Fig. S6).

To reveal the key biological processes mediated by *CsMLO4* in cucumber defense against *C. cassiicola*, we conducted Gene Ontology (GO) and Kyoto Encyclopedia of Genes and Genomes (KEGG) analyses on the DEGs of the WT vs *Csmlo4* and *Csmlo4* vs *Csmlo4*+ groups (Fig. 9D). According to the GO analysis, in the biology process category, organelle organization and oxidation–reduction process were the most common GO terms in the WT vs *Csmlo4* and *Csmlo4* vs *Csmlo4*+ groups, respectively; in the cellular component category, non-membrane-bounded organelle and transcription regulator complex were the most abundant GO terms in the WT vs *Csmlo4* group and *Csmlo4* vs *Csmlo4*+ group, respectively; in the molecular function category, microtubule binding, tubulin binding, and cytoskeletal protein binding were the three GO terms with the highest enrichment in the WT vs *Csmlo4* group, while catalytic activity was the most prominent GO term in the *Csmlo4* vs *Csmlo4*+ group. Furthermore, KEGG analysis demonstrated that three pathways were enriched in the WT vs *Csmlo4* group: carotenoid biosynthesis, alpha-linolenic acid metabolism, and sesquiterpenoid and triterpenoid biosynthesis. In contrast, in the *Csmlo4* vs *Csmlo4*+ group, a total of 10 pathways were enriched, including phenylpropanoid biosynthesis, plant–pathogen interaction, phenylalanine metabolism, cutin, suberine and wax biosynthesis, photosynthesis–antenna proteins, MAPK signaling pathway–plant, glutathione metabolism, selenocompound metabolism, cyanoamino acid metabolism, and flavonoid biosynthesis. The unique metabolic pathways of these two groups indicate the diversity of biological processes involved in *CsMLO4*-mediated cucumber resistance to *C. cassiicola*.

### Transient overexpression of *CsMYB* promotes defense against *C. cassiicola*

As one of the largest transcription factors in plants, the MYB family plays an important role in plant disease control and prevention. Based on RNA-seq, we obtained a potential candidate gene *CsMYB* (CsaV3_5G012310) regulated by *CsMLO4* (Fig. 10). Therefore, we attempted to investigate the role of *CsMYB* in the defense against *C. cassiicola* to further elucidate the molecular mechanism by which *CsMLO4* regulates cucumber resistance to *C. cassiicola*.

**Figure 11:**
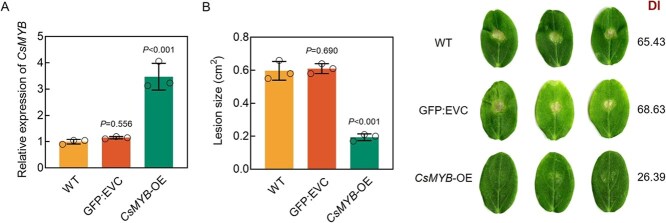
Overexpression of *CsMYB* increases the resistance of cucumber to *C. cassiicola*. (A) Expression levels of *CsMYB* in WT, empty vector control (GFP:EVC), and overexpression (*CsMYB*-OE) cucumbers*.* (B) Disease resistance of WT, GFP:EVC, and *CsMYB*-OE cucumber cotyledons was assessed using phenotypic symptoms, DI statistics, and lesion size at 5 dpi with *C. cassiicola*. Data are means ± SD of three biological replicates per variety. Significance was assessed by the LSD multiple comparison test.

Using cucumber transient transformation technology, we introduced the empty vector control p35S::GFP and the overexpression vector p35S::*CsMYB*-GFP into the cotyledons of WT cucumber seedlings, generating GFP:EVC and *CsMYB*-OE plants, respectively. Compared with WT and GFP:EVC plants, the expression level of *CsMYB* in *CsMYB*-OE plants was significantly increased (Fig. 11A). Five days postinfection with *C. cassiicola*, *CsMYB*-OE plants showed milder symptoms, smaller lesion size, and lower DI than WT and GFP:EVC plants (Fig. 11B), suggesting that overexpression of *CsMYB* enhanced cucumber resistance to *C. cassiicola*.

## Discussion

TLS, caused by *C. cassiicola*, is a severe leaf disease significantly reducing cucumber yield and quality [3]. Despite its impact, the pathogenic mechanisms underlying TLS remain poorly understood, underscoring the need to identify key resistance genes and develop disease-resistant germplasm. In this study, we identified and characterized *CsMLO4*, a novel TLS resistance gene, using a cucumber *Tnt1* retrotransposon insertion mutant library combined with BSA-seq (Fig. 1).

### Unique role of *CsMLO4* in cucumber defense against *C. cassiicola*

*CsMLO4* encodes a protein that has seven transmembrane domains and is localized to the PM, typical of the MLO protein family, and reveals the conserved structure of this group (Fig. S2; Fig. 2B). While MLO proteins are widely known to be susceptible to powdery mildew [7, 33], their roles in resistance to other pathogens remain underexplored. We found that *CsMLO4* positively regulates cucumber defense against *C. cassiicola*, unlike previously reported negative regulators *CsMLO1* and *CsMLO2* [34]. Loss of function and silencing of *CsMLO4* significantly reduced resistance to *C. cassiicola*, while its overexpression enhanced resistance, as evidenced by reduced TLS lesions and increased expression of defense genes *CsPR1*, *CsPR2*, and *CsPR3* (Figs 3, 5, and  7). Unlike previously characterized MLO proteins, which primarily contribute to plant immunity in clades IV and V, CsMLO4 is uniquely positioned within clade III [13, 35]. This distinct classification underscores the specificity and potential novelty of *CsMLO4* in pathogen defense mechanisms.

### Role of ROS in *CsMLO4*-mediated defense against *C. cassiicola* in cucumber

ROS plays a crucial role in the early defense response to pathogen attacks [23]. Rapid accumulation of ROS can effectively inhibit pathogen invasion, serving as a critical component of the plant immune response [26, 27, 36]. In this study, we demonstrated that *CsMLO4* actively regulates cucumber resistance to *C. cassiicola* by promoting ROS accumulation. Specifically, loss of function and silencing of *CsMLO4* resulted in reduced levels of H_2_O_2_ and O_2_^−^ during 0–72 hpi, whereas overexpression of *CsMLO4* resulted in significantly increased ROS levels (Figs 4, 6, and  8). However, excessive ROS accumulation can cause oxidative damage, negatively affecting plant growth and defense [32]. To mitigate this, plants employ antioxidant defense systems, including POD, SOD, and CAT, which are essential for ROS scavenging and maintaining homeostasis. The activities of these enzymes have been strongly linked to improved pathogen resistance in plants [37]. For example, in citrus, the transcription factor CsWRKY65 enhances fruit resistance to *Penicillium digitatum* by increasing SOD, POD, and CAT activity [38].

Consistent with these observations, our study revealed that the activities of SOD, POD, and CAT were markedly reduced in *Csmlo4* mutants compared to WT plants (Fig. 4E). This reduction likely reflects an impaired antioxidant response in the mutants, which may have helped prevent oxidative damage caused by excessive ROS during the early stages of *C. cassiicola* infection. These findings provide insights into how *CsMLO4* mediates defense against *C. cassiicola* by regulating ROS accumulation and homeostasis, further advancing our understanding of the interplay between ROS dynamics and plant immunity.

### Molecular mechanisms of *CsMLO4*-mediated resistance to *C. cassiicola* in cucumber

Further RNA-seq data will help reveal the molecular mechanisms underlying *CsMLO4*-mediated resistance to *C. cassiicola*. Among the identified DEGs, several transcription factors were highlighted, including bHLH, MYB, AP2/ERF, and GATA. These transcription factors are known to function in plant defense against pathogens [39–42], suggesting that they may be critical regulators in the *CsMLO4*-mediated resistance pathway. Functional analysis of *CsMYB* revealed that its overexpression significantly enhanced defense against *C. cassiicola* (Fig. 11). This mode of action is analogous to that of *CsMLO4*, implying that *CsMLO4* may positively regulate the expression of *CsMYB*, thereby promoting cucumber resistance to *C. cassiicola*.

Additionally, RNA-seq revealed two abscisic acid (ABA)-related genes (*AKR* and *CYP707A1*) and one SA-related gene (*BEBT*), which are critical components of plant hormone signaling during immune responses [43, 44]. The observed down-regulation of *CsMLO4* expression by ABA and SA (Fig. S7) suggests that these hormonal pathways could be integral to *CsMLO4*-mediated defense mechanisms, with *AKR*, *CYP707A1*, and *BEBT* serving as potential downstream targets. Beyond transcription factors and hormone signals, DEGs associated with diverse biological processes were identified, including cell wall remodeling, secondary metabolism, light signaling, calcium signaling, growth and development, DNA/protein modifications, transporter proteins, and defense responses (Fig. 10; Table S2). These findings indicate a broad regulatory network involving *CsMLO4* in the cucumber defense against *C. cassiicola*.

Future research should focus on validating the roles of these DEGs and their associated signaling pathways in *CsMLO4*-mediated resistance. These studies will deepen our understanding of the functional framework of *CsMLO4* and also identify novel targets for enhancing cucumber resistance to *C. cassiicola*.

In summary, this study identified *CsMLO4* as a novel resistance gene against TLS in cucumber and demonstrated its crucial role in promoting defense against *C. cassiicola* by regulating ROS accumulation. Furthermore, RNA-seq analysis uncovered several candidate genes and biological processes influenced by *CsMLO4* during the defense response, highlighting its involvement in a complex regulatory network. Furthermore, through transient expression analysis, it was preliminarily proven that *CsMYB*, one of the potential target genes of *CsMLO4*, is a positive regulator of TLS in cucumbers. These findings provide a solid foundation for uncovering the molecular mechanisms of cucumber–TLS interactions and offer a promising genetic target for enhancing disease resistance in cucumbers through advanced breeding and genetic engineering approaches.

## Materials and methods

### Plant materials

The cucumber cultivar CCMC (WT, 24TN902 line, TN183-2 line, *Tnt1* transposon mutant library, TLS-resistant inbred line S06) and the tobacco cultivar *Nicotiana benthamiana* were used in this study. The above plants were cultured at 26°C with a photoperiod of dark (8 hours)/light (16 hours).

### Pathogen inoculation

*Corynespora cassiicola* was retained in the laboratory and propagated on the PDA medium. For punching inoculation, use a punch with a diameter of 5 mm to obtain fungus mass for inoculation of cucumber cotyledons. For spray inoculation, *C. cassiicola* was ‘swept’ into distilled water using a sterile brush and then filtered through gauze to prepare a suspension (2 × 10^5^ spores·ml^−1^) for inoculation of cucumber euphylla.

### Gene cloning

Sample genomic DNA and total RNA were extracted by the *SteadyPure* Plant Genomic DNA Extraction Kit (Accurate Biotechnology, Hunan, China) and the Eastep® Super Total RNA Extraction Kit (Promega, Beijing, China), respectively. Reverse transcription was performed by the *Evo M-MLV* RT for PCR Kit (Accurate Biotechnology, Hunan, China). The corresponding sequences were cloned using gene-specific primers, and the PCR was performed according to the Hieff Canace® Gold High-Fidelity DNA Polymerase (Yeasen, Shanghai, China) protocol. The primers are listed in Table S3.

### Gene expression pattern analysis

Gene expression patterns were analyzed using 2-week-old cucumber seedlings. For euphylla inoculated with *C. cassiicola*, leaves were harvested for 0, 12, 24, 48, and 72 hpi for gene expression analysis in response to *C. cassiicola*; for euphylla sprayed with 1 mmol·l^−1^ SA, 100 μmol·l^−1^ ABA, and distilled water (control), leaves were harvested for 12, 24, and 48 hours postspray for gene expression analysis in response to hormones.

The gene expression patterns of the above samples were evaluated by qRT-PCR, adhering to the SYBR Green Premix *Pro Taq* HS qPCR Kit (Accurate Biotechnology, Hunan, China) protocol in a LightCycler® 480 II (Roche, Rotkreuz, Switzerland), with *CsActin* as the internal control.

### Subcellular localization

The CDS of *CsMLO4* were ligated into the pRI101-GFP vector with a GFP-tag driven by the 35S promoter to construct p35S::*CsMLO4*-GFP, with p35S::GFP as a control. The p35S::*CsMLO4*-GFP, p35S::GFP, and PM marker were introduced into *Agrobacterium tumefaciens* GV3101 strains, respectively, followed by infiltration of the bacterial suspension into *N. benthamiana*. After 2 days of cultivation, GFP and PM marker fluorescence were imaged using a laser confocal microscope (Nikon, A1+, Tokyo, Japan).

### Identification of the TLS resistance-related gene *CsMLO4*

The TLS susceptibility mutant was discovered and studied in the 24TN902 line while screening the cucumber *Tnt1* transposon mutant library in the spring of 2024.

Regional linkage analysis was performed using the variants identified from BSA-seq. Genome resequencing was performed to identify potential *Tnt1* insertion sites, which were subsequently validated by PCR. To genotype in the M_2_ population, we selected four *Tnt1* insertion sites within the candidate interval to design markers. Sequencing was performed by Beijing Biomarker Technology Cooperation (Beijing, China).

### Transient overexpression assay

For *CsMLO4*, the CDS of *CsMLO4* were ligated into the pRI101-GFP vector with a GFP-tag driven by the 35S promoter to construct p35S::*CsMLO4*-GFP. p35S::*CsMLO4*-GFP was introduced into the *A. tumefaciens* EHA105 strains and permeated into 7-day-old WT and *Csmlo4* cucumber seedling cotyledons to generate *CsMLO4*-OE and p35S::*CsMLO4*/*Csmlo4* plants. Five days postinfiltration, the expression levels of *CsMLO4* in WT, p35S::*CsMLO4*/*Csmlo4*, and *CsMLO4*-OE plants were demonstrated by qRT-PCR. The cotyledons at 48 hours of penetration were inoculated with *C. cassiicola* and harvested at 0, 12, 24, 48, and 72 hpi for DAB and NBT staining as well as determination of H_2_O_2_ and O_2_^−^ contents.

For *CsMYB*, the CDS of *CsMYB* were ligated into the pRI101-GFP vector with a GFP-tag driven by the 35S promoter to construct p35S::*CsMYB*-GFP. Overexpression vector p35S::*CsMYB*-GFP and empty vector pRI101-GFP were introduced into the *A. tumefaciens* EHA105 strains and permeated into 7-day-old WT cucumber seedling cotyledons, to generate *CsMYB*-OE and GFP:EVC plants. Five days postinfiltration, the expression levels of *CsMYB* in WT, GFP:EVC, and *CsMYB*-OE plants were demonstrated by qRT-PCR.

Additionally, the cotyledons were harvested at 5 dpi for phenotypic identification, lesion size investigation, and DI statistics.

### VIGS assay

For TRV-mediated VIGS, the specific fragment (281 bp) of *CsMLO4* CDS was recombined into the pTRV2 vector. The recombinant vector pTRV2-*CsMLO4* and the empty vectors pTRV1 and pTRV2 were introduced into the *A. tumefaciens* EHA105 strains. The bacterial suspension of pTRV1 was mixed with pTRV2 and pTRV2-*CsMLO4* in equal volumes to generate TRV:EVC and TRV:*CsMLO4*, respectively, which permeated into 7-day-old cucumber seedling cotyledons. Phenotypic observations and gene expression analyses were performed on cotyledons infiltrated for 9 days. Cotyledons infiltrated for 7 days were inoculated with *C. cassiicola* and harvested at 0, 12, 24, 48, and 72 hpi for DAB and NBT staining as well as determination of H_2_O_2_ and O_2_^−^ contents. The cotyledons were harvested at 5 dpi for resistance assessment, including phenotypic identification, lesion size investigation, and DI statistics.

For CGMMV-mediated VIGS, the specific fragment (281 bp) of *CsMLO4* CDS was inserted into the pV190 vector to generate pV190:*CsMLO4*. pV190:*CsMLO4* and pV190:EVC (empty vector control) were introduced into the *A. tumefaciens* GV3101 strains and then permeated into 2-week-old cucumber seedling cotyledons. Expression analyses were performed on euphylla infiltrated for 39 days to verify gene silencing. Plants infiltrated for 35 days were inoculated with *C. cassiicola*. Phenotypic identification and DI statistics were performed on euphylla at 7 dpi.

### Lesion size investigation and DI statistics

For lesion size investigation, the cross-vertical method was used to measure the diameter of the lesion on the inoculated leaves once each, and the average value was calculated.

For DI statistics, the grading standards and calculation formula of disease class were based on the study by Meng *et al.* [45]. Based on the proportion of the leaf area affected by the pathogen relative to the total leaf area, the severity of the disease was categorized into six grades ranging from 0 to 9. Grade 0 indicates no disease manifestation. Grade 1 indicates that the affected area constitutes less than 5% of the total leaf area. Grade 3 indicates that the affected area ranges from 5% to 30% of the total leaf area. Grade 5 indicates that the affected area ranges from 30% to 50% of the total leaf area. Grade 7 indicates that the affected area ranges from 50% to 75% of the total leaf area. Grade 9 indicates that the affected area ranges from 75% to 100% of the total leaf area. The DI was calculated using the following formula: DI = [Σ (*N* × *D*)/(*H* × *T*)] × 100, where *N* = number of plants in each disease grade, *D* = the disease grade, *H* = the highest disease grade, and *T* = the total number of observed plants.

The resistance grading criteria were as follows: high resistance (DI_0_), 0 < DI ≤ 25; moderate resistance (DI_1_), 25 < DI ≤ 50; moderate susceptibility (DI_2_), 50 < DI ≤ 75; high susceptibility (DI_3_), DI > 75.

At least 30 plants were used in each treatment for lesion size investigation and DI statistics.

### DAB staining

Fresh cucumber leaves were soaked in DAB staining solution (0.1 g of DAB tetrahydrochloride, 5 ml of 0.2 mol·l^−1^ Na_2_HPO_4_, 95 ml of distilled water, and 50 μl of Tween 20), treated under vacuum for 10 minutes, and shaken for 6 hours. Subsequently, the staining solution was poured off, and decolorizing solution (glycerol/glacial acetic acid/ethanol = 1:1:3, v/v/v) was added to a water bath at 100°C until the leaf tissues were transparent, and then photographed.

### NBT staining

Fresh cucumber leaves were soaked in 0.1 mg·ml^−1^ NBT staining solution, treated under vacuum for 10 minutes, and shaken for 6 hours. Subsequently, the staining solution was discarded and heated in a boiling water bath by adding a decolorizing solution (glycerol/glacial acetic acid/ethanol = 1:1:3, v/v/v) until the leaf tissues were transparent, and then photographed.

### Determination of H_2_O_2_ and O_2_^−^ contents

For H_2_O_2_, 0.1 g of the sample was added to 1 ml of precooled acetone for ice bath homogenization. The sample was made up to 1 ml with acetone and centrifuged for 10 minutes. The supernatant was taken to detect the absorbance value at 415 nm, and then the H_2_O_2_ content was calculated according to the fresh weight of the sample. The details of the method were referred to the instructions of the H_2_O_2_ kit (Grace Biotechnology, Suzhou, China).

For O_2_^−^, 0.1 g of the sample was added to 1 ml of the extract for ice bath homogenization, then centrifuged for 10 minutes. The supernatant was aspirated to detect the absorbance value at 540 nm, and then the O_2_^−^ content was calculated based on the fresh weight of the sample, as detailed in the instructions of oxygen free radical kit (Grace Biotechnology, Suzhou, China).

### Determination of SOD, CAT, and POD activities

Of the sample, 0.1 g was added to 1 ml of the extract for ice bath homogenization. After centrifugation for 10 minutes, the supernatant was aspirated to measure the absorbance values at 560, 510, and 470 nm, respectively. Then the activities of SOD, CAT, and POD were calculated respectively according to the fresh weight of the samples. The details of the method were referred to the instructions of the corresponding kits (Grace Biotechnology, Suzhou, China).

### RNA sequencing

RNA-seq was performed on WT and *Csmlo4* cucumber euphylla before and after inoculation with *C. cassiicola* by Illumina high-throughput sequencing platform (Personalbio, Nanjing, China). Clean reads were compared to the Cucumber (Chinese Long) v3 Genome | Cucurbit Genomics Database (CuGenDB) using HISAT2 software. Fragments per kilo bases per million fragments were used to normalize expression levels. DEGs were screened using DESeq software (*P* < 0.05 and |log_2_FoldChange| > 1). Functional annotation of DEGs was performed through GO and KEGG databases, with *P* < 0.05 as the significant enrichment criterion.

### Statistical and bioinformatics analysis

The gene and protein sequences were obtained from the NCBI and CuGenDB database. Transmembrane structural analyses were performed by DeepTMHMM. Conserved domain analysis was carried out with NCBI’s CD-Search. The phylogenetic tree construction was carried out with MEGA. Significance of the difference was calculated by the Duncan (*P* ≤ 0.05) or LSD multiple comparison test of IBM SPSS Statistics 27 software. The transcriptome data were analyzed on the Personalbio Cloud Platform. Each sample underwent three biological replicates.

## Supplementary Material

Web_Material_uhaf225

## Data Availability

The data underlying this article are available in the article and in its online supplementary material.
