# Supplementary material for: A novel MLO protein CsMLO4 plays an essential role in cucumber resistance to target leaf spot
Source: Hortic Res. 2025 Sep 10;12(12):uhaf225. doi: 10.1093/hr/uhaf225 (PMC12679914; doi:10.1093/hr/uhaf225)
Supplement: Web_Material_uhaf225 [file web_material_uhaf225.zip › Supporting Figures.docx]

**Supporting Information**

**A Novel MLO Protein CsMLO4 Plays an Essential Role in Cucumber Resistance to Target Leaf Spot**

Yongbo Yu^1,^†, Xiangnan Meng^1,2,^†, Yang Yu^1,2^, Jiajing He^1^, Yuying Jiang^1^, Jinghang Hong^1^, Na Cui^1,2^, Run Cai^4^, Jan Pan^3^, Junsong Pan^4,^*, Haiyan Fan^1,2,^*

^1^College of Bioscience and Biotechnology, Shenyang Agricultural University, Shenyang, 110866, China

^2^Key Laboratory of Protected Horticulture of Ministry of Education, Shenyang Agricultural University, Shenyang, 110866, China

^3^College of Horticulture, Shenyang Agricultural University, Shenyang, 110866, China

^4^College of Agriculture and Biology, Shanghai Jiao Tong University, Shanghai, 200240, China

The following Supporting Figures is available for this article:

**Figure S1.** Loss-of-function of *CsMLO4* enhances the susceptibility of cucumber to *Corynespora cassiicola*.

**Figure S2.** Sequence analysis of CsMLO4.

**Figure S3.** Phylogenetic analysis of MLO4 homologous proteins from different plant species.

**Figure S4.** Expression analysis of *CsMLO4* in WT, p35S::*CsMLO4*/*Csmlo4*, and overexpression (*CsMLO4*-OE) cucumber cotyledons inoculated with *C. cassiicola*.

**Figure S5.** Silencing of *CsMLO4* increases the susceptibility of cucumber to *C. cassiicola*.

**Figure S6.** Expression analysis of differentially expressed genes (DEGs) in WT and *Csmlo4* plants before and after inoculation with *C. cassiicola*.

**Figure S7.** Expression analysis of *CsMLO4* in cucumbers treated with distilled water (Con.), salicylic acid (SA) and abscisic acid (ABA) at different time-points.


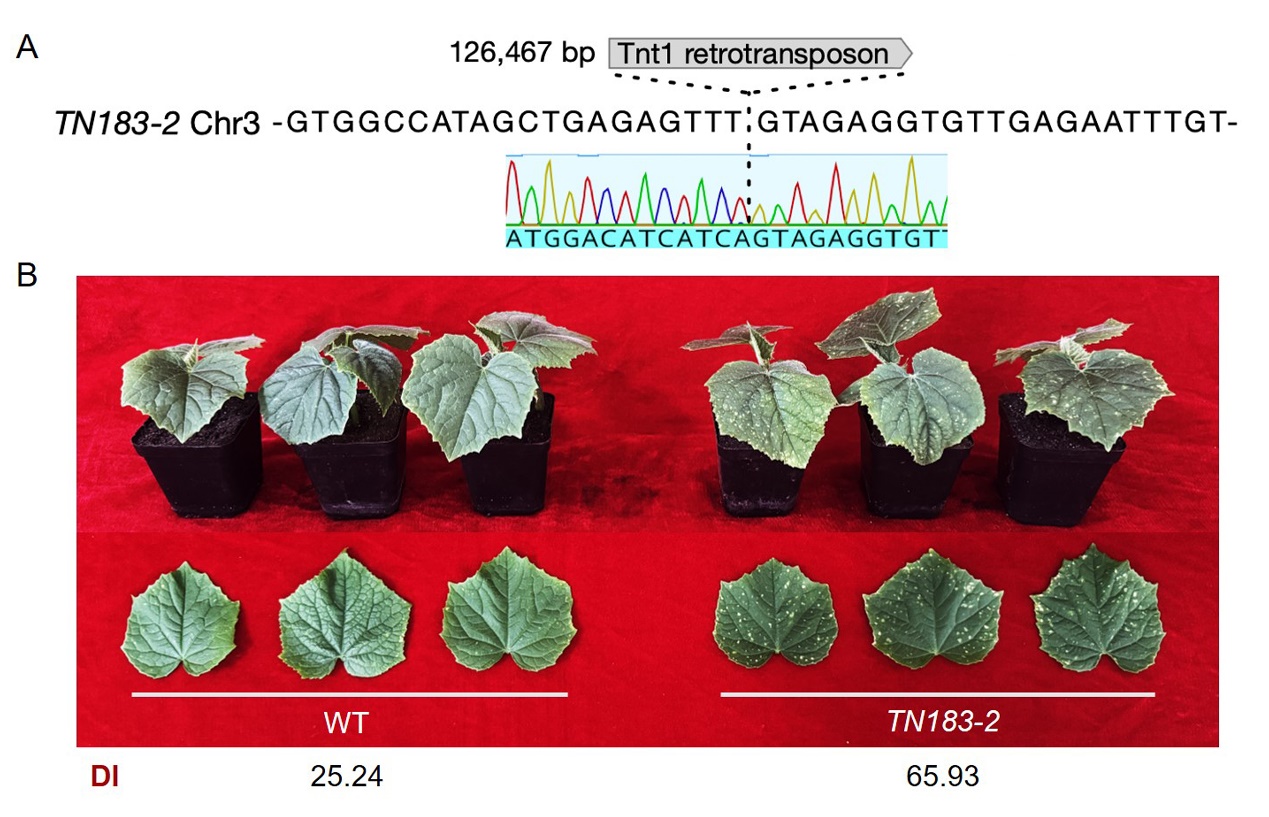
**Figure S1.** Loss-of-function of *CsMLO4* enhances the susceptibility of cucumber to *Corynespora cassiicola*. **(A)** The insertion site of the *Tnt1* retrotransposon within the candidate gene *Csa3G000160* (*CsMLO4*) in the TN183-2 line. **(B)** Disease resistance of wild-type (WT) and loss-of-function of *CsMLO4* mutant line TN183-2 leaves was assessed using phenotypic symptoms and disease index (DI) statistics at 7 days post-inoculation (dpi) with *C. cassiicola*.


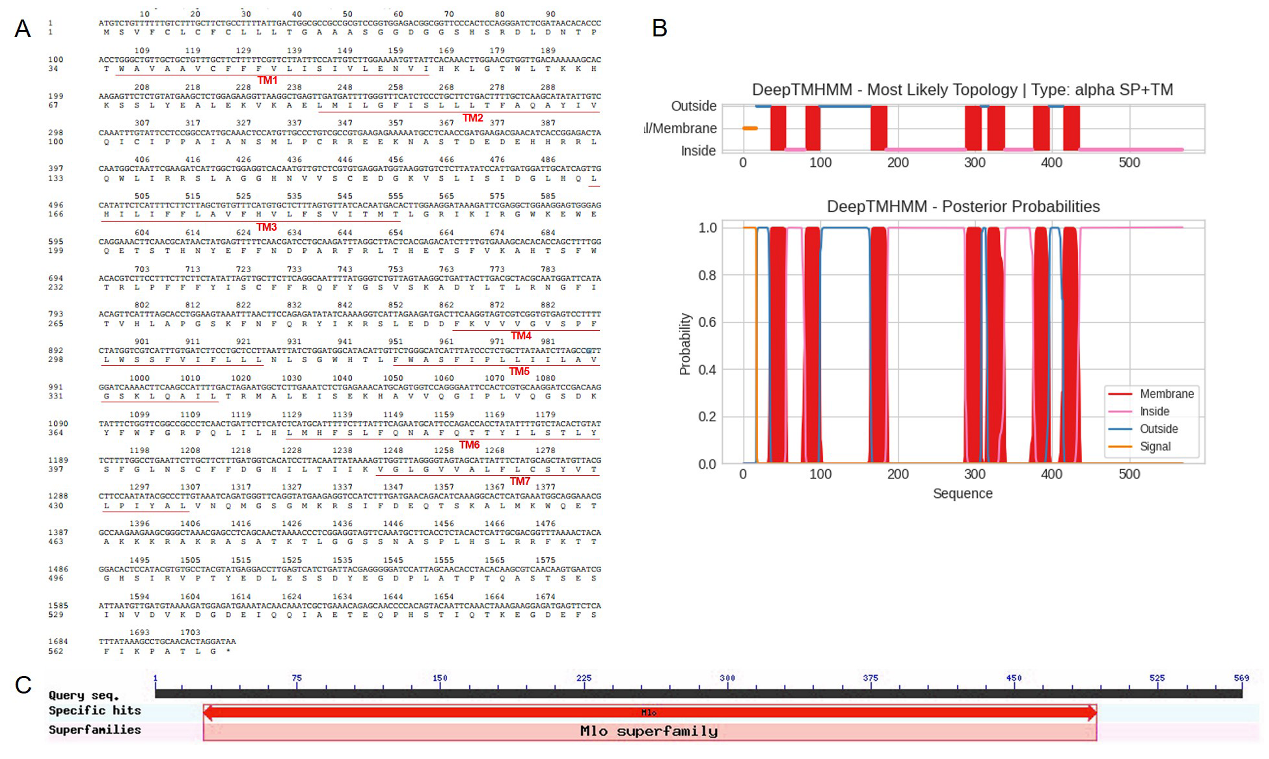


**Figure S2.** Sequence analysis of CsMLO4. **(A)** Amino acid sequence of CsMLO4. TM1–TM7, seven transmembrane domains of CsMLO4. **(B)** Analysis of the transmembrane structure of CsMLO4. **(C)** The conserved domain of CsMLO4.


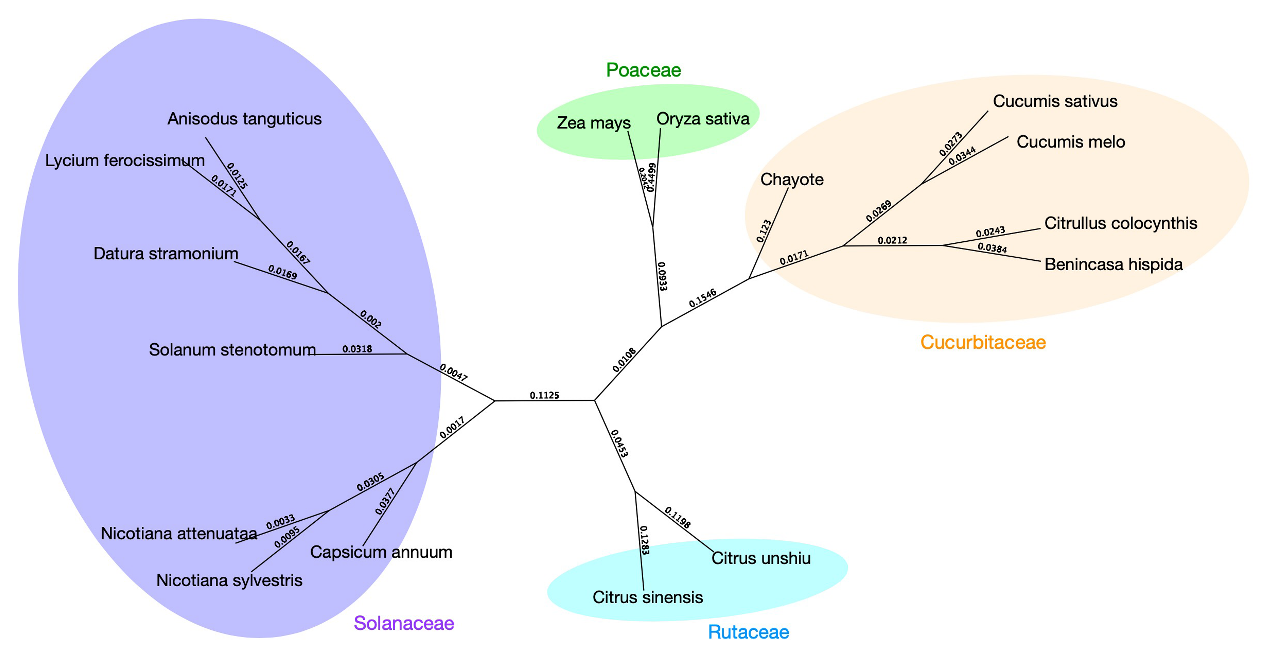


**Figure S3.** Phylogenetic analysis of MLO4 homologous proteins from different plant species. The phylogenetic tree depicted in the illustration was generated based on the sequences of MLO homologous proteins from 16 plant species. The sequences of MLO homologous proteins were obtained from the NCBI and Cucurbit Genomics Database (CuGenDB) database. Protein login numbers: *Anisodus tanguticus*, KAK4360470; *Benincasa hispida*, XP_038904161; *Citrus unshiu*, GAY59431; *Citrus sinensis*, KAH9662261; *Citrullus colocynthis*, CAK9321354; *Cucumis melo*, XP_008448445; *Cucumis sativus*, XP_004146181; *Capsicum annuum*, XP_016558628; *Datura stramonium*, MCD7454059; *Lycium ferocissimum*, XP_059280800; *Nicotiana attenuata*, XP_019231107; *Nicotiana sylvestris*, XP_009790030; *Oryza sativa*, LOC_Os03g03700; Chayote, Sed0025426; *Solanum stenotomum*, XP_049385336; *Zea mays*, Zm00001eb271870.


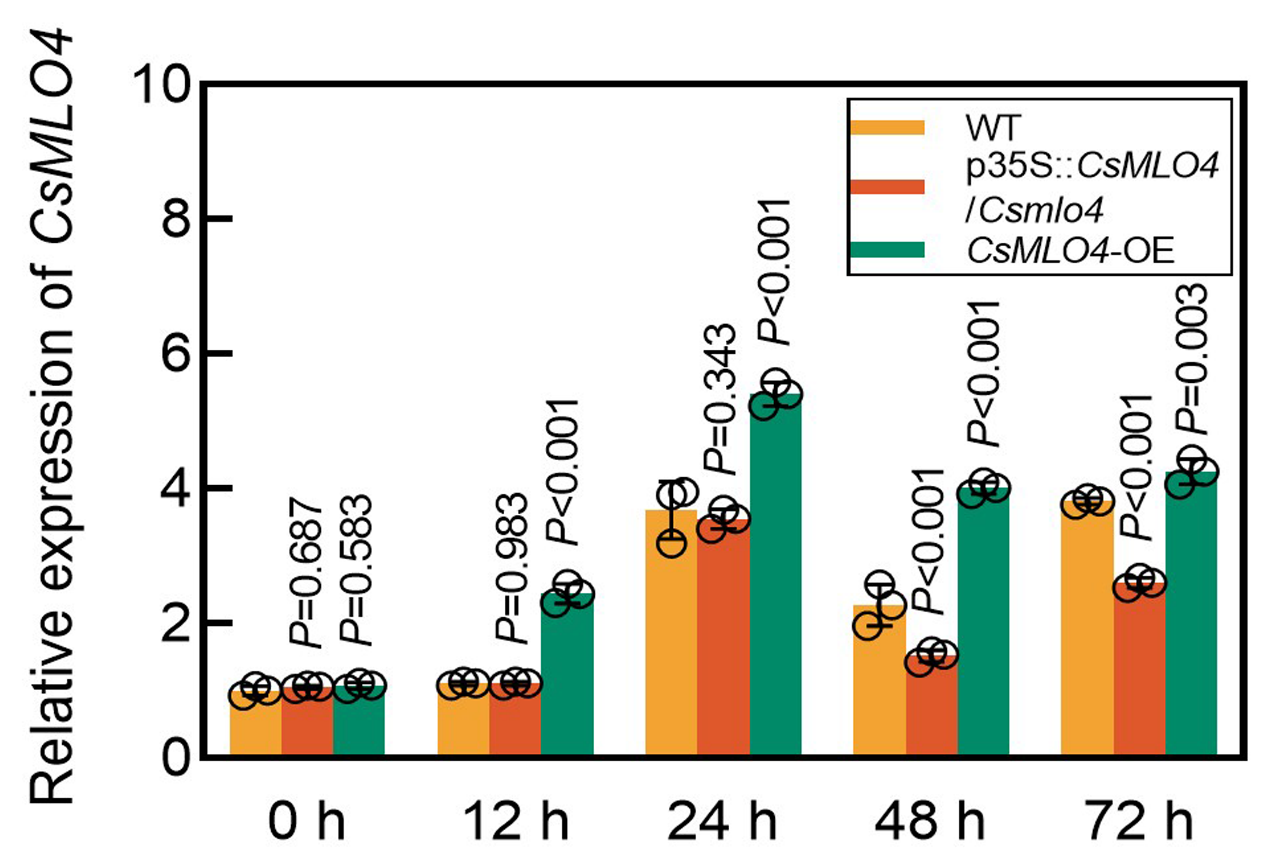


**Figure S4.** Expression analysis of *CsMLO4* in WT, p35S::*CsMLO4*/*Csmlo4*, and overexpression (*CsMLO4*-OE) cucumber cotyledons inoculated with *C. cassiicola*. Data are means ± SD of three biological replicates per variety. Significance was assessed by the LSD multiple comparison test.


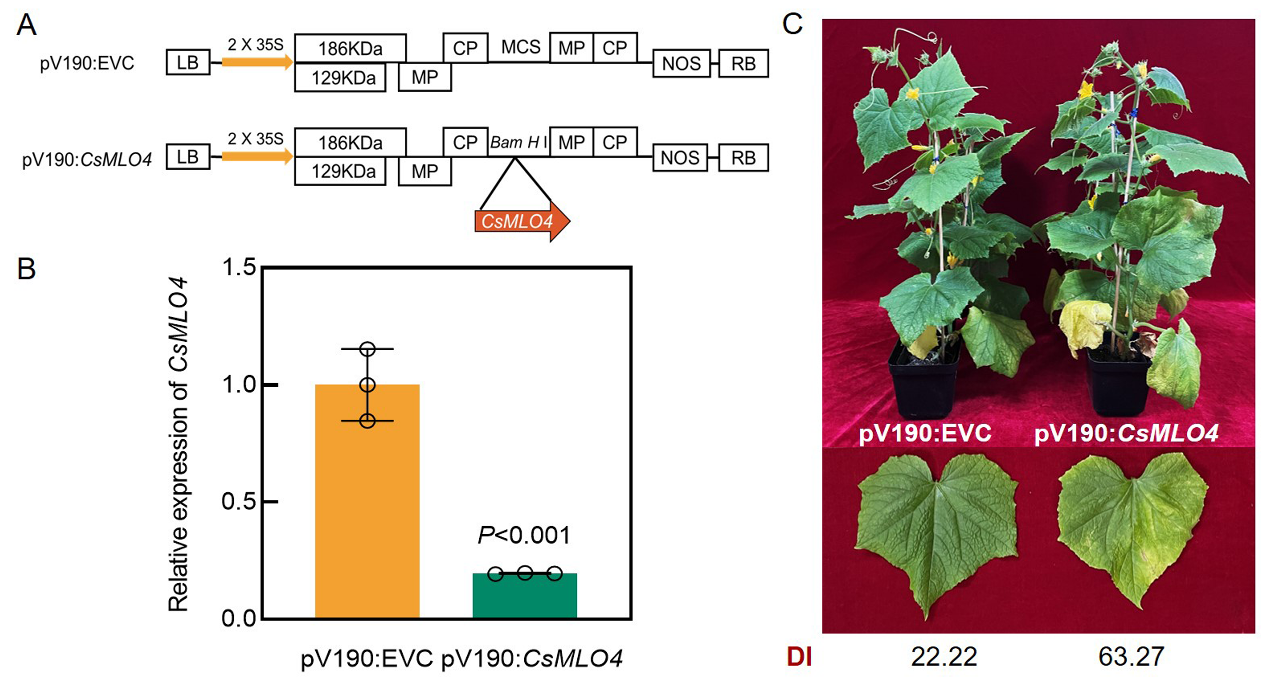


**Figure S5.** Silencing of *CsMLO4* increases the susceptibility of cucumber to *C. cassiicola*. **(A)** Schematic of pV190:EVC and pV190:*CsMLO4* vectors. **(B)** Expression levels of *CsMLO4* in pV190:EVC and pV190:*CsMLO4* cucumbers*.* Data are means ± SD of three biological replicates per variety. Significance was assessed by the LSD multiple comparison test. **(C)** Disease resistance of pV190:EVC and pV190:*CsMLO4* cucumber leaves was assessed using phenotypic symptoms and DI statistics at 7 dpi with *C. cassiicola*.


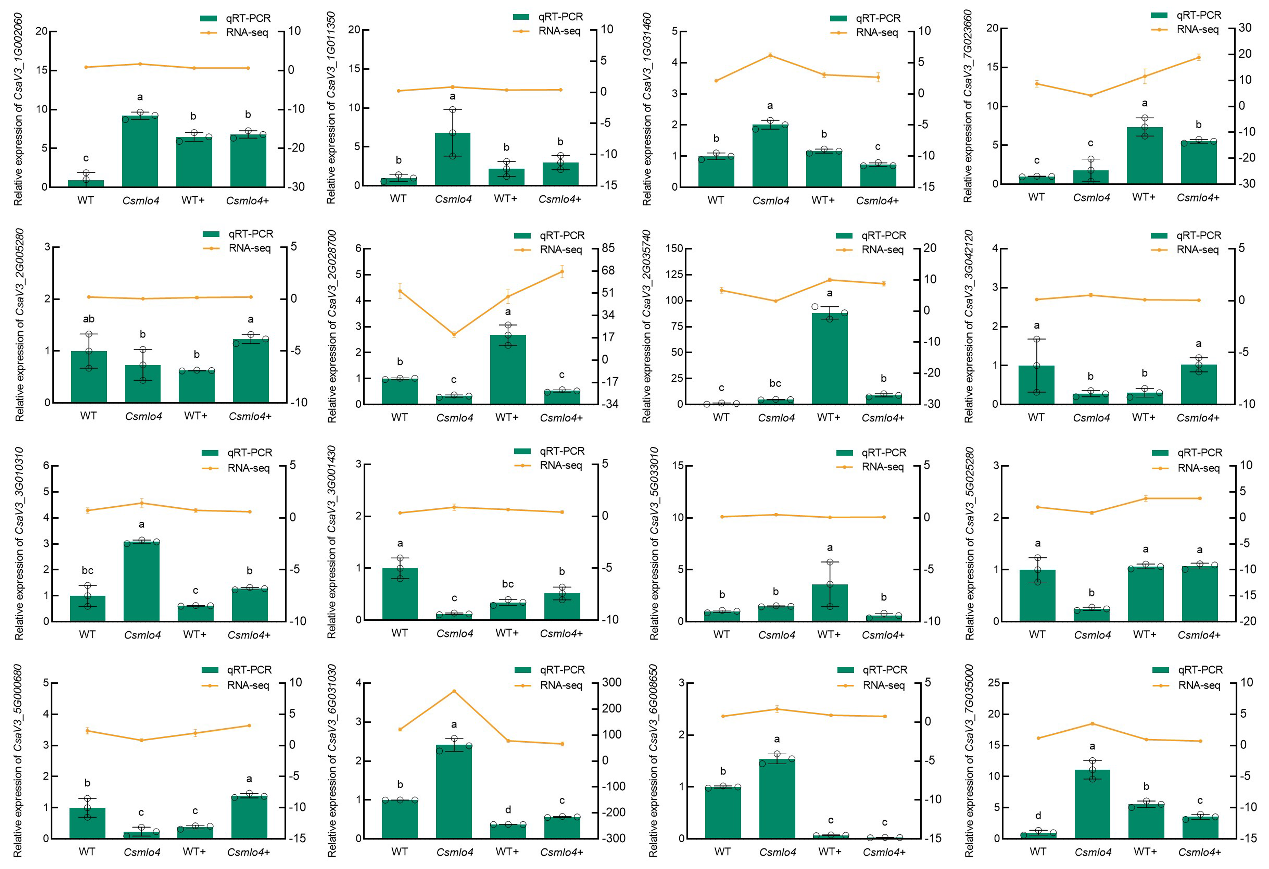


**Figure S6.** Expression analysis of differentially expressed genes (DEGs) in WT and *Csmlo4* plants before and after inoculation with *C. cassiicola*. Data are means ± SD of three biological replicates per variety. Significance was assessed by the Duncan multiple comparison test (*P* ≤ 0.05). “+” means that the sample was infected with *C. cassicola* for 24 h.


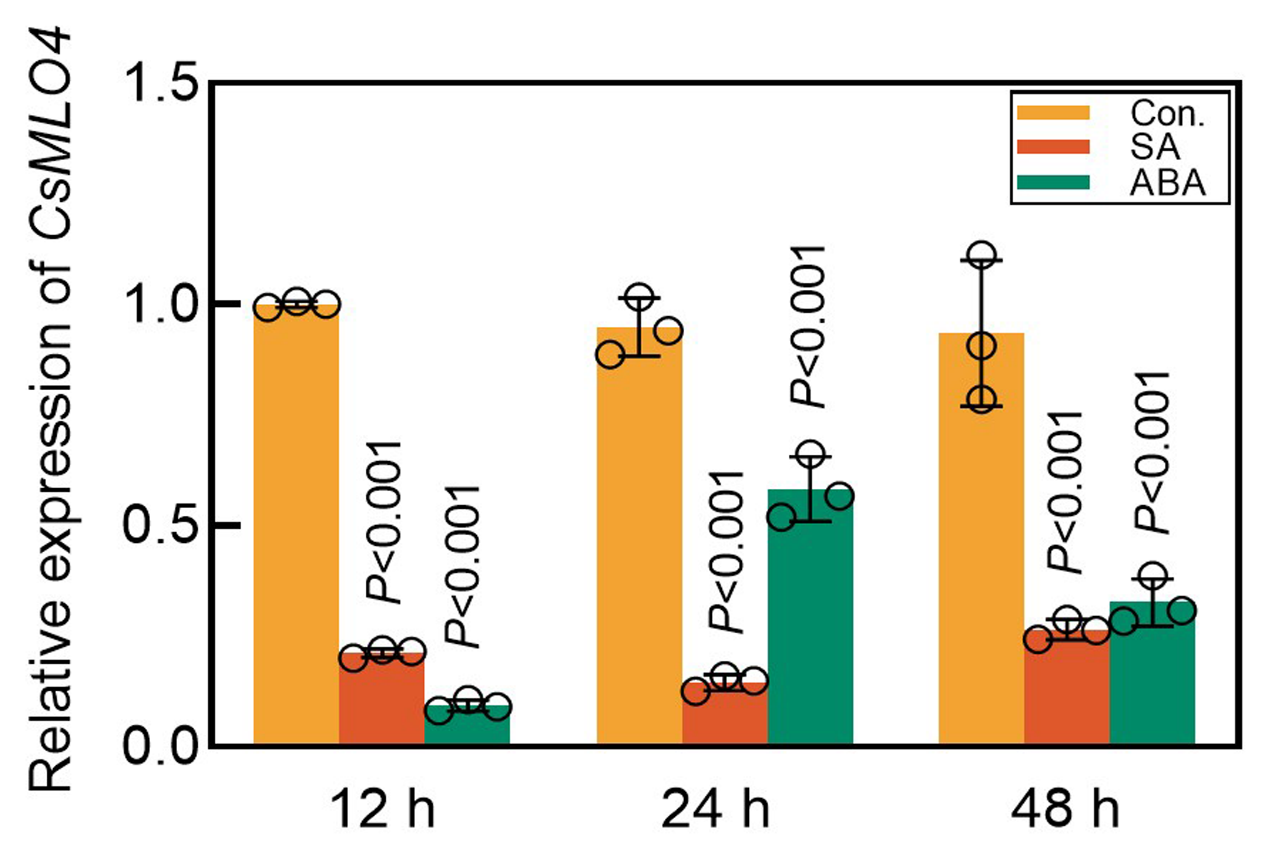


**Figure S7.** Expression analysis of *CsMLO4* in cucumbers treated with distilled water (Con.), salicylic acid (SA) and abscisic acid (ABA) at different time-points. Data are means ± SD of three biological replicates per variety. Significance was assessed by the LSD multiple comparison test.
